# New Foci of Buruli Ulcer, Angola and Democratic Republic of Congo 

**DOI:** 10.3201/eid1411.071649

**Published:** 2008-11

**Authors:** Kapay Kibadi, Mbutu Panda, Jean-Jacques Muyembe Tamfum, Alexandra G. Fraga, Adhemar Longatto Filho, Gladys Anyo, Jorge Pedrosa, Yoshinori Nakazawa, Patrick Suykerbuyk, Wayne M. Meyers, Françoise Portaels

**Affiliations:** Institute of Tropical Medicine, Antwerp, Belgium (K. Kibadi, G. Anyo, P. Suykerbuyk, F. Portaels); University of Kinshasa, Kinshasa, Democratic Republic of Congo (K. Kibadi, M. Panda); Institut National de Recherche Biomédicale, Kinshasa (K. Kibadi, J.-J. M. Tamfum); University of Minho, Braga, Portugal (A.G. Fraga, A.L. Filho, J. Pedrosa); University of Kansas, Lawrence, Kansas, USA (Y. Nakazawa); Armed Forces Institute of Pathology, Washington, DC, USA (W.M. Meyers)

**Keywords:** Mycobacterium ulcerans, Buruli ulcer, Kwango/Cuango River, Democratic Republic of Congo, Angola, artisanal alluvial mining, dispatch

## Abstract

We report 3 patients with laboratory-confirmed Buruli ulcer in Kafufu/Luremo, Angola, and Kasongo-Lunda, Democratic Republic of Congo. These villages are near the Kwango/Cuango River, which flows through both countries. Further investigation of artisanal alluvial mining as a risk factor for Buruli ulcer is recommended.

Buruli ulcer (BU), which is caused by the bacterium *Mycobacterium ulcerans*, is an indolent necrotizing disease of skin, subcutaneous tissue, and bone. BU is the third most common mycobacterial disease of humans, after tuberculosis and leprosy (*1*,*2*). Africa is the most affected continent, particularly in its tropical, central, and western regions (*1*).

BU was first reported in the Democratic Republic of Congo (DRC) in 1950 (*1*). The disease has been reported in 5 of 11 provinces in DRC (Lower Congo, Bandundu, Maniema, Katanga, and South-Kivu) (*3*).

BU was first reported in Angola in Caxito, Bengo Province, in 1998 (Figure) (*4*). Reports of BU in newly arrived Angolan refugees at Kimpese (Lower Congo) since the 1960s (*5*) suggest that Angola has long been an area endemic for BU. However, no cases have been reported along the Kwango/Cuango River in DRC or Angola. This river, known as the Kwango River in DRC and the Cuango River in Angola, is the boundary between Angola and DRC from Luremo to Kasongo-Lunda (Bandundu Province) (Figure).

**Figure:**
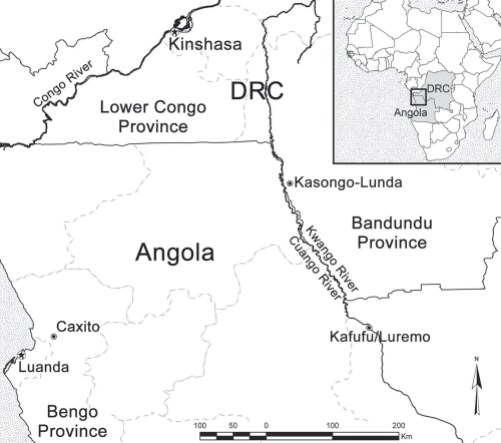
Locations in Democratic Republic of Congo (DRC) (Kasongo-Lunda) and Angola (Kafufu/Luremo) where 3 patients with Buruli ulcer were detected.

This study describes 3 laboratory-confirmed cases of BU. These cases were most likely acquired near the Kwango/Cuango River.

## The Study

We studied 3 patients suspected of having BU who were admitted to the Dr Lelo Medical Center in Kinshasa (patient 1) and the Mother Teresa Buruli Ulcer Treatment Center in Kinshasa (patients 2 and 3). The study was reviewed and approved by the ethics committee of the Institute of Tropical Medicine, Antwerp, and the Public Health School of the Kinshasa University, Kinshasa, Ministère de l’Enseignement. The 3 patients provided verbal consent to participate in the study. Patients 1 and 2 were men 30 and 28 years of age, respectively, and patient 3 was a girl 13 years of age.

Characteristics of the patients are shown in the Table. Laboratory tests were performed on surgically excised tissues and exudates according to World Health Organization (WHO) recommendations (*6*). Patients were treated with rifampin and streptomycin for 12 weeks according to WHO recommendations (*7*). Four weeks after the beginning of treatment, surgical debridement was performed, followed by split-skin grafting. The patients were followed up at the 2 treatment centers and were considered cured when the lesions had completely healed.

All patients were residents of Kinshasa (DRC) and had no contacts with areas endemic for BU before traveling to areas along the Kwango/Cuango River where the BU skin lesions first appeared. However, all patients had frequent contact through alluvial diamond mining (patients 1 and 2) or domestic activities (patient 3) along the Kwango/Cuango River in DRC (Kasongo-Lunda) or in Angola (Kafufu/Luremo) (Figure).

Patients reported that their lesions had started 2–2.5 years earlier as nodules that later ulcerated. These patients were first treated locally near the Kwango/Cuango River by traditional healers and with 2% Dakin fluid (sodium hypochlorite solution) to cleanse the wounds. These measures were unsuccessful, and the patients returned to Kinshasa for treatment at the 2 medical centers, where they were admitted in September 2004 (patient 1), June 2005 (patient 2), and July 2005 (patient 3).

On admission, all 3 patients had large ulcers (150–896 cm^2^). Patient 1 had an ulcer on the right thigh, patient 2 on the right arm, and patient 3 on the left leg (Table). BU was confirmed by Ziehl-Neelsen staining for acid-fast bacilli and a positive IS*2404* PCR result (Table). Cultures remained negative after incubation for 12 months at 32°C. For patient 3, BU was also confirmed by histopathologic analysis performed before treatment. A specimen showed a predominantly neutrophilic inflammatory infiltrate near extensive areas of necrosis associated with calcification and clumps of extracellular acid-fast bacilli.

**Table:** Characteristics of 3 Buruli ulcer patients infected along the Kwango/Cuango River, DRC and Angola*

Characteristic	Patient 1	Patient 2	Patient 3
Age, y, sex	30, M	28, M	13, F
Origin	Kinshasa, DRC	Kinshasa, DRC	Kinshasa, DRC
Location where infected	Kafufu/Luremo, Angola	Kafufu/Luremo, Angola	Kasongo-Lunda, DRC
Patient delay,† y	2	2.5	2.5
Date of first symptoms	2002 Oct	2003 Jan	2003 Jan
Date care was sought	2004 Sep	2005 Jun	2005 Jul
Lesion			
Type	Ulcer	Ulcer	Ulcer
Size, cm^2^	320	150	896
Site	Right thigh	Right arm	Left leg
Test results			
Ziehl-Neelsen staining	+	+	+
Culture	–	–	–
IS*2404* PCR	+	+	+
Histopathologic changes	ND	ND	Extensive areas of necrosis with clumps of AFB
Duration of hospitalization, mo	3	6	7
Follow-up period with no relapse, mo	42	30	28
Outcome	Cured	Cured	Cured

*DRC, Democratic Republic of Congo; +, positive; –, negative; IS, insertion sequence; ND, not done; AFB, acid-fast bacilli. †Time between appearance of first signs or symptoms and care being sought at a medical center.

The 3 patients were considered cured after 3, 6, and 7 months, respectively, of hospitalization. No relapses were observed after follow-up periods of 42, 30, and 28 months, respectively.

## Conclusions

BU patients in our study had advanced disease with ulcers >10 cm in diameter. They were cured by treatment with antimicrobial drugs and surgery in accordance with WHO recommendations for treatment of BU (*6*,*7*). The patients were most likely infected during alluvial mining and use of water from the Kwango/Cuango River for domestic activities. Artisanal diamond mining in alluvial deposits along river banks consists of informal digging with basic equipment (often with unprotected hands and feet). Miners often work for long periods extracting diamonds from alluvial deposits along river banks. In Angola, mining areas are located in swamps that border the Kwango/Cuango River. Water sources used for domestic purposes along this slow-flowing river are unprotected, and proper hygienic procedures are lacking.

Epidemiologic studies have established a close association of BU and wetlands, especially those with slow-flowing or stagnant water (ponds, backwaters, and swamps) (*8*–*10*). In Uganda and in Benin, use of unprotected sources of water for domestic purposes increased the risk for contracting BU (*10*–*12*). Environmental factors, including poor hygienic conditions, along the Kwango/Cuango River make this region an area of high risk for contracting BU (*10*,*13*).

Studies have linked increased incidence of BU to human-made modifications such as expanded agricultural activities, deforestation, or construction of dams (*14*). Activities of both large mining enterprises and individual miners are responsible for environmental changes that may play a role in increased incidence of BU. Diamond-mining pits may become pools of stagnant waters that are a dangerous source of waterborne diseases.

In the 1950s in the Belgian Congo (now DRC), several cases of BU were reported in alluvial gold miners working in the mining camp of Kakerifu between the Nzoro and Kibali Rivers (*1*). Currently, in the gold-mining area of Amansie West District in Ghana, many BU infections occur among workers in contiguous alluvial mining operations (*15*). Diamond mines along the Cuango River in Angola may have influenced the emergence of BU cases along this river basin. However, whether the Cuango River floodplain is a region endemic for BU or if this region became endemic after diamond-mining activities is not known.

The frequency of BU in Angola is not documented partly because of political changes after the country’s independence in 1975. Surveys are urgently needed to determine the endemicity of BU in Angola. Our findings emphasize the need for further investigation of diamond, gold, and any other gemstone mining as a risk factor for contracting BU, particularly in West and Central Africa, where mining is common. All areas along the Kwango/Cuango River in DRC and Angola should be investigated for foci of BU. The association of artisanal alluvial mining with BU draws attention to a disease that further diminishes the quality of life of persons who are already living under the precarious circumstances experienced by those who mine diamonds.
